# Do nomadic lactobacilli fit as potential vaginal probiotics? The answer lies in a successful selective multi-step and scoring approach

**DOI:** 10.1186/s12934-023-02030-4

**Published:** 2023-02-11

**Authors:** Claudia Cappello, Marta Acin-Albiac, Daniela Pinto, Andrea Polo, Pasquale Filannino, Fabio Rinaldi, Marco Gobbetti, Raffaella Di Cagno

**Affiliations:** 1 Faculty of Science and Technology, Free University of Bolzano, Bolzano, Italy; 2 Human Microbiome Advanced Project, Research & Development, Milan, Italy; 3 Department of Soil, Plant and Food Science, University of Bari Aldo Moro, Bari, Italy

**Keywords:** Vaginal microbiota, Vaginal ecosystem, Nomadic lactobacilli, Probiotics, Pathogens inhibition screening

## Abstract

**Background:**

The goal of this study was to create a multi-strain probiotic gel that would foster a lactobacilli-dominated vaginal microbiota in pregnant women and ensure appropriate eubiosis for the newborn. Nomadic lactobacilli (95 strains), mostly isolated from food sources, were preliminarily screened for functional traits before being characterized for their capability to inhibit the two vaginal pathogens *Streptococcus agalactiae* and *Candida albicans*, which may lead to adverse pregnancy-related outcomes. Eight best-performing strains were chosen and furtherly investigated for their ability to produce biofilm. Lastly, the two selected potential probiotic candidates were analyzed in vitro for their ability to reduce the inflammation caused by *C. albicans* infection on the reconstituted human vaginal epithelium (HVE).

**Results:**

*Lactiplantibacillu*s *plantarum* produced both isomers of lactic acid, while *Lacticaseibacillus paracasei* produced only l-isomer. The production of hydrogen peroxide was strain-dependent, with the highest concentrations found within *Lact. paracasei* strains. The auto-aggregation capacity and hydrophobicity traits were species-independent. *S. agalactiae* 88II3 was strongly inhibited both at pH 7.0 and 4.0, whereas the inhibition of *C. albicans* UNIBZ54 was less frequent. Overall, *L. plantarum* strains had the highest pathogen inhibition and functional scoring. *L. plantarum* C5 and POM1, which were selected as potential probiotic candidates also based on their ability to form biofilms, were able to counteract the inflammation process caused by *C. albicans* infection in the HVE model.

**Conclusions:**

Our multi-step and cumulative scoring-based approach was proven successful in mining and highlighting the probiotic potential of two nomadic lactobacilli strains (*L. plantarum* C5 and POM1), being applicable to preserve and improve human vaginal health.

## Background

The human vaginal microbiome is a key determinant of vaginal health. The vagina ecosystem is often dominated by highly adapted lactobacilli. Vaginal microbiota can be clustered into five community state types (CSTs). Four of these CSTs are dominated by *Lactobacillus crispatus* (CST-I), *Lactobacillus iners* (CST-III), *Lactobacillus gasseri* (CST-II) or *Lactobacillus jensenii* (CST-V), while CST-IV is characterized by strict and facultative anaerobes not belonging to *Lactobacillus* genus [1]. The presence of *Lactobacillus* spp. is correlated with a healthy vaginal microbiome, whereas the CTS-IV is associated with a status of dysbiosis and a consequently higher risk of infections and obstetric complications. Functional traits of lactobacilli involve the production of lactic acid, hydrogen peroxide, and synthesis of bacteriocins as a response to the imbalance of the vaginal microbiome, host protection against vaginal pathogens, promotion of immunomodulation mechanisms by triggering the innate immunity system, and stimulation of anti-inflammatory mechanisms [2–5]. Infections of the host such as bacterial vaginosis (BV), urinary tract infections, yeast vaginitis, and sexually transmitted diseases, like human immunodeficiency virus, are all prevented in large part by the microorganisms that colonize the vaginal environment [6]. Aerobic vaginitis defined by disruption in *Lactobacillus* dominance is accompanied by more extreme inflammatory changes than BV and the presence of mainly aerobic enteric commensals or pathogens, including *Streptococcus agalactiae* (Group B *Streptococcus* [GBS]). *S. agalactiae* is a member of the commensal microbiota of the human intestinal and genitourinary tracts and rarely causes infections in healthy adults. Infrequently it may cause morbidity in the elderly, pregnant women, and patients with underlying predisposing factors. Pregnancy-related maternal GBS colonization during birth is linked to newborn pneumonia, meningitis, and sepsis [7]. *Candida albicans* is a widespread fungus that lives in human mucosa and a few other environmental reservoirs. The human mouth, vagina, and stomach colonization normally begins in infancy, mostly after vaginal delivery or breastfeeding. The vagina is the primary mucocutaneous surface affected by *C. albicans*, resulting in vulvovaginal candidiasis (VVC) [8].

Vaginal microbiota transits among different CSTs during a woman's lifespan, being *Lactobacillus*-dominated, are more common among reproductive age and, particularly, in a pregnant woman. In addition, other lactobacilli may also be found*,* especially nomadic species like *Lactiplantibacillus plantarum* and *Lacticaseibacillus casei* group [2, 6]. A nomadic lifestyle is formally defined as a dynamic, generalist way of life of a species that involves both environmental and host niches, with no signs of specialization [9, 10]. Consequently, nomadic lactobacilli are found in contrasting environments, from vertebrates and invertebrate hosts to fermented vegetable and dairy products [11–15]. These bacteria did not undergo a reductive evolution strategy and thus their large genomes encompass increased metabolic flexibility, which enables them to thrive in diverse environments [9, 10, 16]. *L. plantarum* exemplifies the paradigm of the nomadic lifestyle and possesses one of the largest genomes among lactobacilli (3.3 Mbp). Strains isolated from different environments could reshape their phenotype expression towards a common direction under the same environmental condition [17]. In the same way, *Lacticaseibacillus rhamnosus* and *Lact. casei* genotypes and phenotypes do not correlate properly with their source of isolation [18–21]. Due to their ability to thrive under contrasting conditions when compared to host-adapted lactobacilli*,* nomadic lactobacilli hold a huge potential as probiotics capable of positively modulating several aspects of human health [22].

Hence, the aim of this study is the selection of nomadic lactobacilli to be used as vaginal probiotics. A multistep approach was followed, where the first step encompassed a wise panel of assays for functional traits and pathogens' inhibition using a custom scoring procedure to select the most promising strains. Then, eight candidate strains were further investigated for biofilm production. Subsequently, the two final promising candidate strains were included in a gel formulation prototype alone or as a binary combination. The ability of these prototypes to reduce the expression of the genes involved in the inflammatory cascade using the reconstituted human vaginal epithelium (HVE) infected with *C. albicans* was evaluated.

## Results

### Lactic acid isomers and hydrogen peroxide quantification

**Fig. 1:**
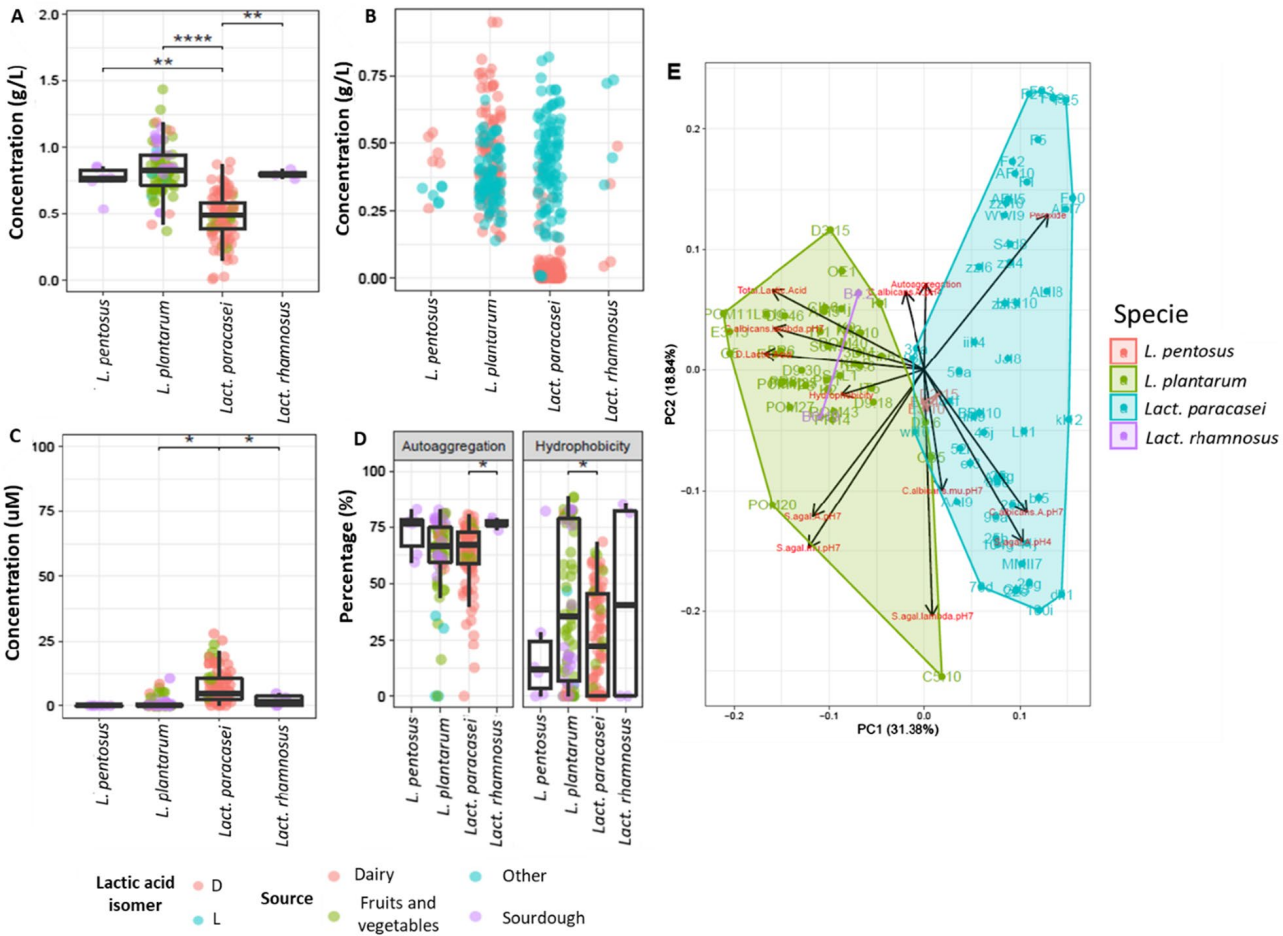
Functional assays results. Functional assays profiling, categorized by strain, of total lactic acid (**A**), lactic acid isomers (**B**), peroxide production (**C**), and auto-aggregation and hydrophobicity (**D**) characterization of nomadic lactobacilli strains after 18–24 h at 37 °C of incubation in MRS. Principal component analysis of data gathered during the first screening (**E**). Dunn test significant statistical differences after FDR correction between groups indicated as follows: * (P < 0.05), ** (P < 0.005), *** (P < 0.0005), **** (P < 0.00005)

The capacity of lactobacilli to produce lactic acid highly varied at species and strain level (Fig. 1A). The highest value of total lactic acid was found for *L. plantarum* POM1 (1.28 ± 0.21 g L^−1^), followed by *L. plantarum* C5 (1.17 ± 0.16 g L^−1^) and D3.15 (1.11 ± 0.06 g L^−1^). Within the *L. pentosus* species, E1.4 strain showed the highest total lactic acid yield (0.82 ± 0. 06 g L^−1^). *Lact. rhamnosus* B4.2 and B6.19 produced almost equal quantities (0.82 ± 0.03 and 0.78 ± 0.02 g L^−1^, respectively). Overall, the production of total lactic acid from *Lact. paracasei* strains was quite low, with the highest amount produced from *Lact. paracasei* 93j (0.83 ± 0.09 g L^−1^).

A significant difference (P < 0.005) was observed between *L. pentosus* and *Lact. paracasei*, and between *Lact. paracasei* and *Lact. rhamnosus*. Moreover *L. plantarum* and *Lact. paracasei* showed a significant difference with P < 0.00005. Further investigation of lactic acid production revealed different patterns of lactic acid isomer production (Fig. 1B). Among *L. plantarum* strains, POM1 produced the highest amount of d-lactate (0.86 ± 0.12 g L^−1^), followed by C5 (0.83 ± 0.17 g L^−1^), and P1 (0.74 ± 0.10 g L^−1^). The production of d-lactate by *L. pentosus* species ranged from 0.48 ± 0.07 g L^−1^ (strain E1.4) to 0.40 ± 0.20 g L^−1^ (strain D2.15). The two *Lact. rhamnosus* strains showed an opposite behavior where *Lact. rhamnosus* B4.2 produced 0.42 ± 0.10 g L^−1^ of d-lactate, whereas *Lact. rhamnosus* B6.19 produced 0.05 ± 0.01 g L^−1^. Almost no strains of *Lact. paracasei* produced d-lactate, with eight strains failing to produce both isomers. The highest l-lactate yields came mostly from the *Lact. paracasei* group, where *Lact. paracasei* 84f showed the highest production (0.76 ± 0.09 g L^−1^). After it came the l-lactate yields from *L. plantarum* strains, with the highest yield found for *L. plantarum* AFI (0.51 ± 0.21 g L^−1^) and the lowest for *L. plantarum* CB5 (0.21 ± 0.02 g L^−1^). *Lact. rhamnosus* B6.19 and B4.2 produced 0.73 ± 0.01 and 0.40 ± 0.07 g L^−1^ of l-lactate, respectively. Within the *L. pentosus* species, the l-lactate highest yield was found for the E1.4 strain (0.34 ± 0.01 g L^−1^), and the lowest for *L. pentosus* D2.15 (0.29 ± 0.02 g L^−1^).

H_2_O_2_ production also showed high variability at species and strains level (Fig. 1C). Overall, *Lact. paracasei* produced the highest amounts of H_2_O_2_ ranging from 0.72 ± 1.02 (25 h) to 22.44 ± 2.07 (ALII8) μM, whereas *Lact. paracasei* 22e was the only one within this species unable to produce H_2_O_2_. No H_2_O_2_ was produced from the strains belonging to *L. pentosus* species. Most of the *L. plantarum* examined did not produce H_2_O_2_, with *L. plantarum* D2.6 (6.07 ± 6.28 μM) having the highest production within the species. *Lact. rhamnosus* B4.2 and B6.19 strains produced 2.45 ± 3.20 and 1.59 ± 2.24 μM, respectively. Based on statistical analysis at the species level, a significant difference (P < 0.05) was observed between *L. plantarum* and *Lact. paracasei*, and between *Lact. paracasei* and *Lact. rhamnosus*.

### Auto-aggregation capacity and hydrophobicity characteristics

The results from auto-aggregation and hydrophobicity assays were species- and especially strain-dependent (Fig. 1D). A significant difference (P < 0.05) was observed only between *Lact. paracasei* and *Lact. rhamnosus* species. However, within the same species some strains were better performing than others. Approximately 60% of lactobacilli strains isolated from various sources and belonging to different species, showed an auto-aggregation capacity above the average value (63.71 ± 4.18%). The highest aggregation capacity was reached by *L. plantarum* D9.46 (81.77 ± 1.01%). The capability of the bacteria to potentially adhere to the epithelial cells was further investigated through the characterization of cell wall hydrophobicity (Fig. 1D). *L. plantarum* S6w5 had the highest hydrophobicity capacity (88.40 ± 0.29%). All the other strains were almost equally distributed around the average value, showing a value lower (41.1% of all the strains) or higher (43.2% of all the strains) than the average (31.5 ± 8.7%) value. Only a few strains (15.8%), belonging to different species and isolated from different sources, did not show any hydrophobicity characteristics. A significant difference (P < 0.05) was observed between *L. plantarum* and *Lact. paracasei*.

### Pathogen growth inhibition screening

**Fig. 2:**
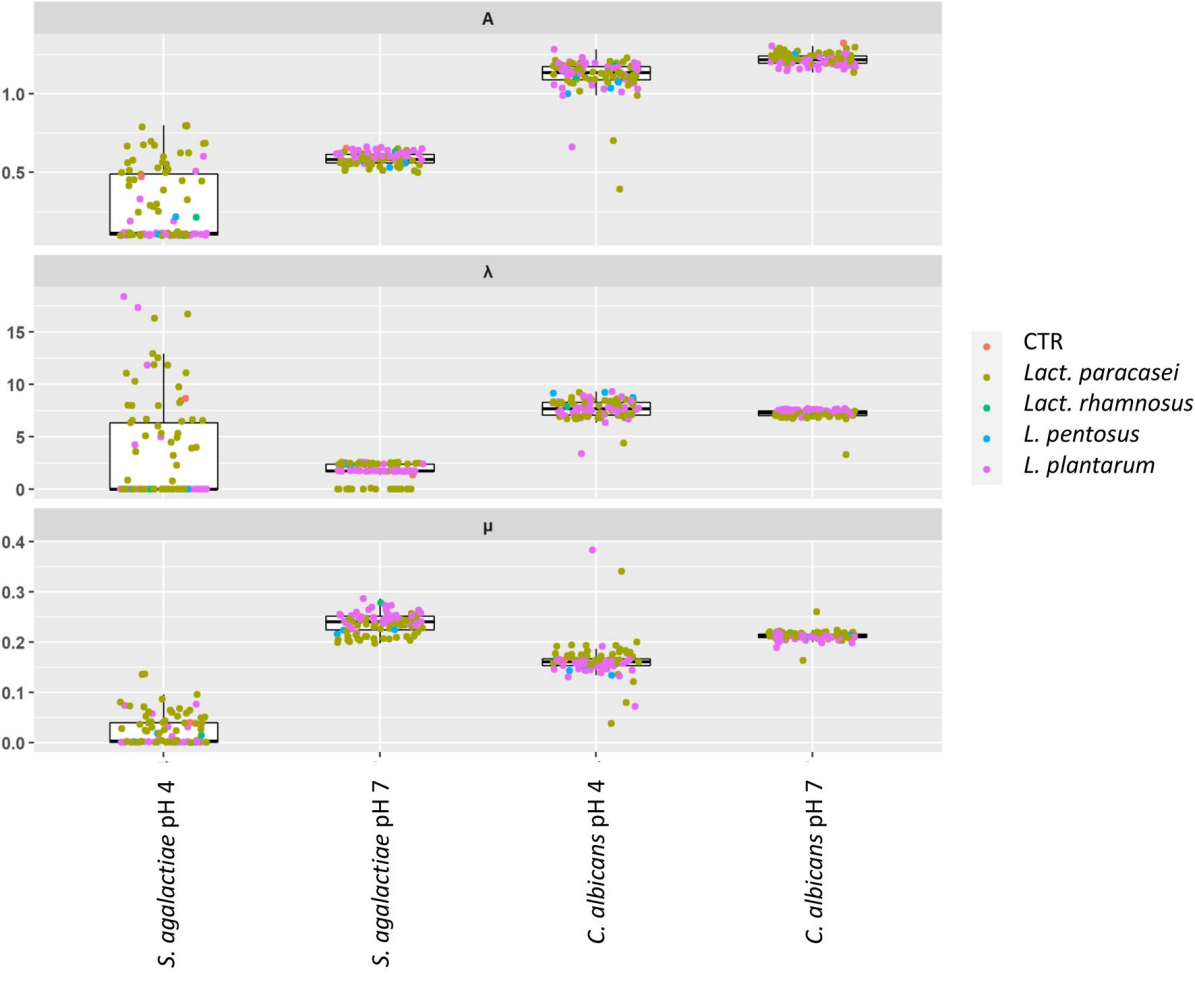
Growth inhibition parameters of *Candida albicans* and *Streptococcus agalactiae* from cell free supernatants kept at original acidic pH (pH ca. 4) or adjusted to neutral pH (pH 7) for all the tested strains and divided by species, compared to the control condition (CRT) of growth of *C. albicans* and *S. agalactiae *

Growth kinetics of *S. agalactiae* 88II3 and *C. albicans* UNIBZ54 were assessed in the presence of cell-free supernatants (CFSs) from all *Lactobacillus* strains and compared against the control conditions (Fig. 2). The inhibitory activity was tested using CFSs at the original pH of ca. 4.0 (o-CFSs) or neutralized CFSs at pH 7 (n-CFSs), to exclude pH-dependent effects. *S. agalactiae* 88II3 grown at pH 7.0 as a control condition reached a maximum absorbance (*A*) of 0.62 ± 0.03, with the lag phase (*λ*) 1.34 ± 0.72 h, and growth rate (*μ*) 0.24 ± 0.02 h^−1^. At pH ca. 4.0, lower *A* (0.47 ± 0.05), longer *λ* (8.67 ± 3.98 h), and smaller* μ* (0.04 ± 0.01 h^−1^) were reached.

Three common patterns of inhibition of *S. agalactiae* 88II3 were observed with n-CFSs, while no common pattern of inhibition was observed with o-CFSs. n-CFSs from seventy lactobacilli strains caused a statistically significant (P < 0.05) reduction of the *A* value when compared to the control condition at pH 7.0. n-CFSs from *Lact. paracasei* HHI10 had the strongest repressive effect on *S. agalactiae* 88II3 in terms of *A* (0.50 ± 0.02). n-CFSs from 25 strains most of which belonged to *L. plantarum* species, did not inhibit *S. agalactiae* 88II3 in terms of *A.* Most of the n-CFSs were collected from *Lact. paracasei* significantly (P < 0.05) extended the lag phase (*λ*) of *S. agalactiae* 88II3, with the highest *λ* value detected when *S. agalactiae* 88II3 was grown with n-CFS from *Lact. paracasei* 104 g (2.58 ± 0.02 h). When *S. agalactiae* 88II3 was cultured with n-CFSs from sixteen *Lact. paracasei* strains no lag phase was observed. Negative values of *λ* can be interpreted biologically as the pathogen starting to grow right after inoculation. Significant (P < 0.05) reduction of *μ* at pH 7.0 was found mostly with n-CFSs collected from *Lact. paracasei* strains, with the lowest (P < 0.05) value for *Lact. paracasei* F23 (0.20 ± 0.00). By monitoring the *A* parameter, complete inhibition of *S. agalactiae* 88II3 growth was found during culturing with o-CFSs from *L. plantarum* (36 strains), *Lact. paracasei* (20), *L. pentosus* (3), and *Lact. rhamnosus* (2). *C. albicans* UNIBZ54 cultured at pH 7.0 as a control condition reached a maximum *A* of 1.31 ± 0.02, with a *λ* of 6.86 ± 0.30 h, and *μ* of 0.21 ± 0.01 h^−1^. At pH 4.0 as the control condition, *C. albicans* UNIBZ54 reached lower *A* (1.18 ± 0.08), longer *λ* (8.22 ± 1.26 h), and smaller* μ* (0.16 ± 0.02 h^−1^). Eight n-CFSs that were obtained from *L. plantarum* (1 strain) and *Lact. paracasei* (7 strains), led to a higher *A* parameter when compared to the control condition at pH 7.0, while 87 strains had a repressive effect (P < 0.05) on the *A* parameter. The n-CFS that was obtained from *Lact. paracasei* wI10 had the strongest inhibition effect in terms of *A* at pH 7.0 (1.13 ± 0.14). n-CFSs from 19 strains, mostly *L. plantarum*, extended the lag phase (*λ* parameter), with the longest value reached with *L. plantarum* 1LS16 (7.68 ± 0.06 h). No significant (P > 0.05) reduction of the *μ* parameter of *C. albicans* UNIBZ54 was observed at pH 7.0. Lower significant inhibition of the growth curve of *C. albicans* UNIBZ54 was observed with o-CFSs under acidic condition (pH 4). Inhibition was obtained from only 12 o-CFSs collected from *Lact. paracasei* (5), *L. plantarum* (6), and *L. pentosus* (1). The lowest value of *A* was reached with o-CFSs from *Lact. paracasei* 41j (0.39 ± 0.17). Only five o-CFSs that were collected from 5 strains of *L. pentosus* (3) and *Lact. paracasei* (2) affected the *λ* value of *C. albicans* UNIBZ54. The highest value of *λ* was detected during incubation with o-CFS from *L. plantarum* D2.6 (9.33 ± 0.18 h). No significant inhibition on the *μ* parameter for *C. albicans* was attributable to the o-CFSs under acidic condition (pH ca. 4.0). To evaluate the differences among all the strains and the overall characteristics investigated, we carried out a principal component analysis (PCA) (Fig. 1E). *L. plantarum* differ from *Lact. paracasei* in their functional and pathogen inhibition traits of interest in vaginal health. Furthermore, particularly interesting is the position of *L. plantarum* POM1 and C5 in the corner of the PCA. These two strains differentiate also from other standalone *L. plantarum* strains.

### Candidate selection approach and scoring procedure

A scoring procedure was set for both the functional and the pathogen inhibition assays for the selection of the best-performing strains through the definition of a cumulative score (see “Methods” section). Overall, the best performing assays for the selection were the d-lactic acid production and the hydrophobicity characteristics, where a total of 45 CFSs showed a relative score of 1 or 2, meaning that the value is either between Q2 and Q3, or higher than Q3. For the H_2_O_2_ production and auto-aggregation evaluation, a total of 44 CFSs showed a relative score equal to 1 or 2, and lastly total lactic acid production, where only 9 strains showed positive results (Table 1).

**Table 1:** Single or double hit of inhibition of *Candida albicans* and *Streptococcus agalactiae* from cell free supernatants kept at original acidic pH (o-CFS) or adjusted to neutral pH (n-CFS) for all the tested strains and divided by species

	*Candida albicans*				*Streptococcus agalactiae*			
	Single hit		Double hit		Single hit		Double hit	
Species	o-CFS	n-CFS	o-CFS	n-CFS	o-CFS	n-CFS	o-CFS	n-CFS
*Lact. paracasei*	5	42	0	1	20	4	0	45
*L. pentosus*	2	3	0	0	3	0	0	3
*L. plantarum*	6	23	0	16	36	18	0	4
*Lact. rhamnosus*	0	0	0	2	2	0	0	0
Grand Total	13	68	0	19	61	22	0	52

The total number of strains categorized per species whose cell-free supernatants at the original acidic pH (o-CFSs) and CFSs at neutral pH (n-CFSs) showed a single and double hit of inhibition on the growth of each pathogen. o-CFS and n-CFS had a significant effect on *A* or *λ* of *C. albicans* UNIBZ54, but never on its *μ*, thus resulting in a single hit of inhibition (Table 1). On the other side, some n-CFS had a significant effect on more than one growth parameter of *S. agalactiae* 88II3 at pH 7.0, giving a double hit of inhibition (Table 1). Moreover, some o-CFS had a remarkable effect on *A* of *S. agalactiae* 88II3 at acidic pH (pH ca. 4.0) (Table 1). Based on their pathogen inhibition and functional assays score (Table 2), eight best-performing *Lactobacillus* strains were selected: *L. plantarum* OE1, C5, D3.15, 1LS16, POM1, E3.8 and D2.6, and *Lact. paracasei* 41j.

**Table 2:** Scoring overview for the first eight best-performing lactobacilli

Specie	Sub-source of isolation	Code	Pathogen inhibition scoring	Functional scoring	Cumulative scoring
*L. plantarum*	Carrot	C5	5	8	13
*L. plantarum*	Olives	OE1	9	4	13
*L. plantarum*	Sourdough	E3.8	10	3	13
*L. plantarum*	Sourdough	D2.6	6	6	12
*L. plantarum*	Sourdough	D3.15	6	5	11
*L. plantarum*	Tomato	POM1	5	6	11
*Lact. paracasei*	Milk	41j	6	5	11
*L. plantarum*	Pineapple	1LS16	5	5	10

### Biofilm imaging by confocal laser scanning microscopy

**Fig. 3:**
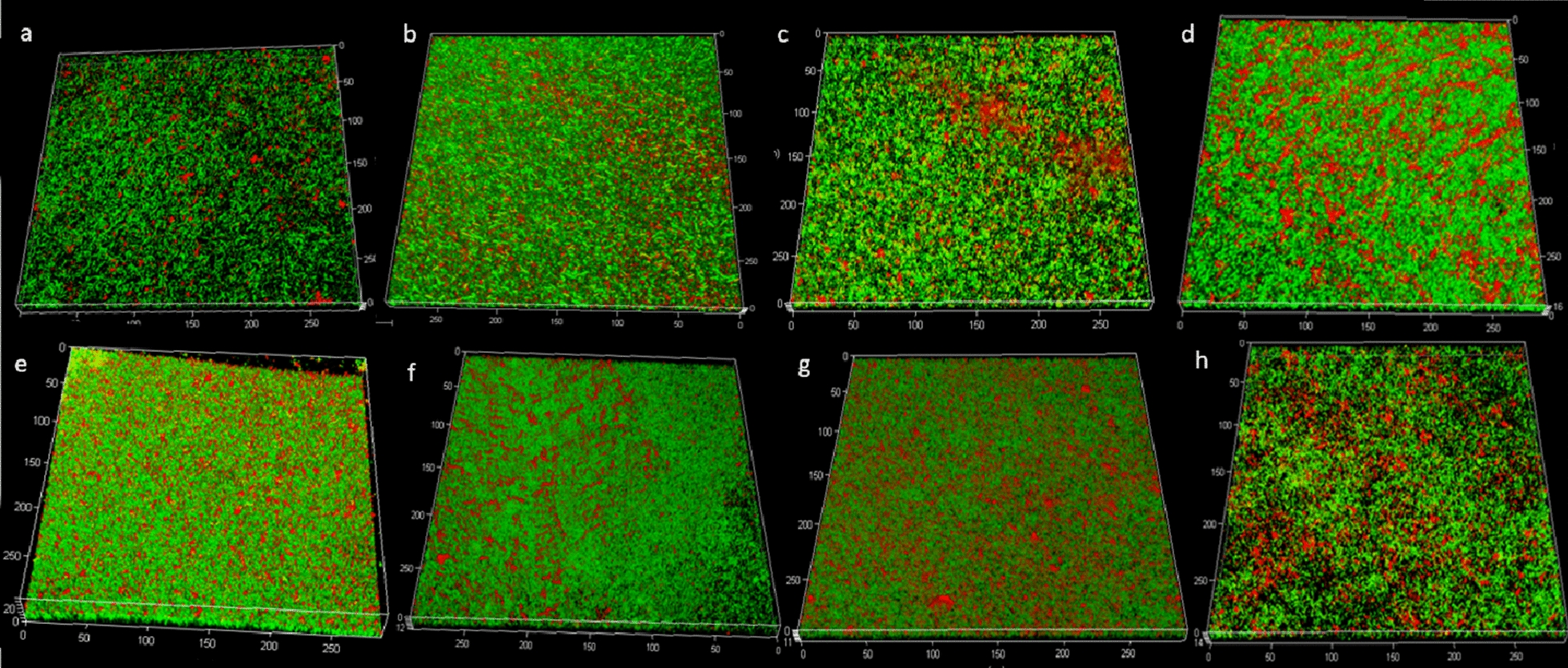
Three dimensional images from biofilms observed under the confocal laser scanning microscopy. Biofilm produced by *L. plantarum* C5 (**a**), *L. plantarum* POM1 (**b**), *L. plantarum* 1LS16 (**c**), *L. plantarum* D3.15 (**d**), *L. plantarum* OE1 (**e**), *L. plantarum* D6.6 (**f**), *L. plantarum* E3.8 (**g**), *Lact. paracasei* 41j (**h**). Metabolically active cells (green fluorescence) and biofilm exopolysaccharides in the extracellular polymeric matrix (red fluorescence) are shown in the same panel. Bars represent 50 μm; units of the x, y, and z axes are μm. Images are representatives of three biological replicates analyzed in triplicate

Three-dimensional images of the biofilm colonies grown on membrane filters were collected through CLSM (confocal laser scanning microscopy) observations of the mature biofilms resulting from the 8 selected strains after 48 h of incubation. All biofilms appeared as widespread agglomerates of sessile cells (green fluorescence) embedded in exopolysaccharides (EPS) (red fluorescence) (Fig. 3). Biofilm architecture was strain-dependent, with a lower quantity of red fluorescence visible in the images captured from *L. plantarum* E3.8 and *L. plantarum* POM1.

### Biofilm growth and extracellular matrix characterization

Biomass, cell density, and extracellular matrix (ECM) (protein, extracellular DNA (eDNA), and total saccharides) content from the mature biofilms of the eight selected strains formed after 48 h of incubation are shown in Table 3. The highest biofilm formation in terms of biomass was achieved by *L. plantarum* POM1 (36.20 ± 12.02 mg), whereas *L. plantarum* C5 led to the lowest one (11.30 ± 1.70 mg). In terms of cell density and ECM protein content, there was no statistically significant difference (P > 0.05) among the strains. The eDNA concentration ranged between a minimum for *Lact. paracasei* 41j (0.59 ± 0.00 µg mg^−1^) to a maximum for *L. plantarum* C5 (1.93 ± 0.00 µg mg^−1^) (P < 0.05). The highest production of EPS was found in ECMs extracted from the biofilm of *L. plantarum* C5 (80.11 ± 0.02 µg mg^−1^), being significantly (P < 0.05) higher than EPS extracted from *L. plantarum* D2.6 (35.00 ± 0.00 µg mg^−1^), *L. plantarum* POM1 (26.11 ± 0.00 µg mg^−1^), *L. plantarum* E3.8 (25.09 ± 0.01 µg mg^−1^), *L. plantarum* 1LS16 (22.44 ± 0.00 µg mg^−1^), and *Lact. paracasei* 41j (17.91 ± 0.02 µg mg^−1^) (P < 0.05). Taking into consideration all these results, two strains, *L. plantarum* C5 and POM1, were further investigated for more specific analysis to understand their behavior in the vaginal environment. However, also *Lact. paracasei* 41j, considering its metabolic traits, may be included in the formulation.

**Table 3:** Biofilm characterization

Strain	Biomass (mg)	Cell density (Log CFU mL^−1^)	eDNA (µg mg^−1^)	Proteins (µg mg^−1^)	EPS (µg mg^−1^)
*L. plantarum* C5	11.30 ± 1.70^b^	9.22 ± 0.27^a^	1.93 ± 0.38^a^	6.87 ± 1.27^a^	80.11 ± 16.98^a^
*L. plantarum* POM1	36.20 ± 12.02^a^	10.13 ± 0.22^a^	0.62 ± 0.10^ab^	1.72 ± 0.36^a^	26.11 ± 1.12^b^
*L. plantarum* 1LS16	34.70 ± 4.81^ab^	9.28 ± 0.15^a^	0.66 ± 0.24^ab^	2.33 ± 0.06^a^	22.44 ± 0.58^b^
*L. plantarum* D3.15	24.15 ± 5.59^ab^	9.40 ± 0.24^a^	1.03 ± 0.21^ab^	3.50 ± 0.16^a^	38.07 ± 5.00^ab^
*L. plantarum* OE1	16.35 ± 9.40^ab^	9.54 ± 0.48^a^	1.16 ± 0.22^ab^	4.77 ± 0.03^a^	44.11 ± 10.84^ab^
*L. plantarum* D2.6	30.85 ± 0.49^ab^	9.56 ± 0.02^a^	0.86 ± 0.34^ab^	2.66 ± 0.02^a^	35.00 ± 1.64^b^
*L. plantarum* E3.8	34.50 ± 1.70^ab^	9.55 ± 0.35^a^	0.75 ± 0.02^ab^	2.45 ± 0.06^a^	25.08 ± 4.79^b^
*Lact. paracasei* 41j	26.80 ± 4.81^ab^	9.44 ± 0.04^a^	0.59 ± 0.18^b^	3.28 ± 0.39^a^	17.91 ± 20.87^b^

Biomass (mg), cell density (Log CFU mL^−1^), and ECMs (eDNA, proteins, and exopolysaccharide concentration, expressed as µg/mg of the total biomass) characterization of the newly formed biofilm, and reported as mean ± standard deviation [a–b Means within the columns with different letters are significantly different (P < 0.05)]

### Evaluation of CFSs preservation of HVE metabolic activity after infection with* C. albicans*

Compared to the negative control, *C. albicans* UNIBZ54 without the addition of CFS (positive control) sharply decreased the metabolic activity of the HVE (25.91 ± 0.78%) according to the MTT assay (Table 4).

**Table 4:** Percentage of metabolic activity of human vaginal epithelium model

Treatment	Viability (%)
Negative control	100.00 ± 0.16^a^
Positive control	25.91 ± 0.78^c^
*L. plantarum* C5	56.78 ± 11.09^b^
*L. plantarum* POM1	64.94 ± 5.24^b^
Combination (*L. plantarum* C5 + POM1)	71.55 ± 9.85^b^

Negative control is the untreated epithelium, positive control is the epithelium infected with *Candida albicans*. *L. plantarum* C5,* L. plantarum* POM1, and C5 + POM1 are the epithelium models infected with the pathogen and then treated with the gel containing the respective strain or the combination of both. Data represent the mean ± standard deviation [a–c Means with different letters are significantly different (P < 0.05)]

Generally, both *L. plantarum* C5 and POM1 strains were able to prevent HVE metabolic activity loss, both when cultured in single or in combination. In detail, the metabolic activity of the infected HVE treated with a gel containing *L. plantarum* C5 and *L. plantarum* POM1 reached 56.78 ± 11.09% and 64.94 ± 5.24%, respectively, with a statistically (P < 0.05) significant difference from the viability of cells of positive control. The combination of the two strains showed a metabolic activity of 71.55 ± 9.85%, demonstrating a synergistic effect of nomadic lactobacilli, still significantly different (P < 0.05) than the positive control.

### Gene expression profiling

**Fig. 4:**
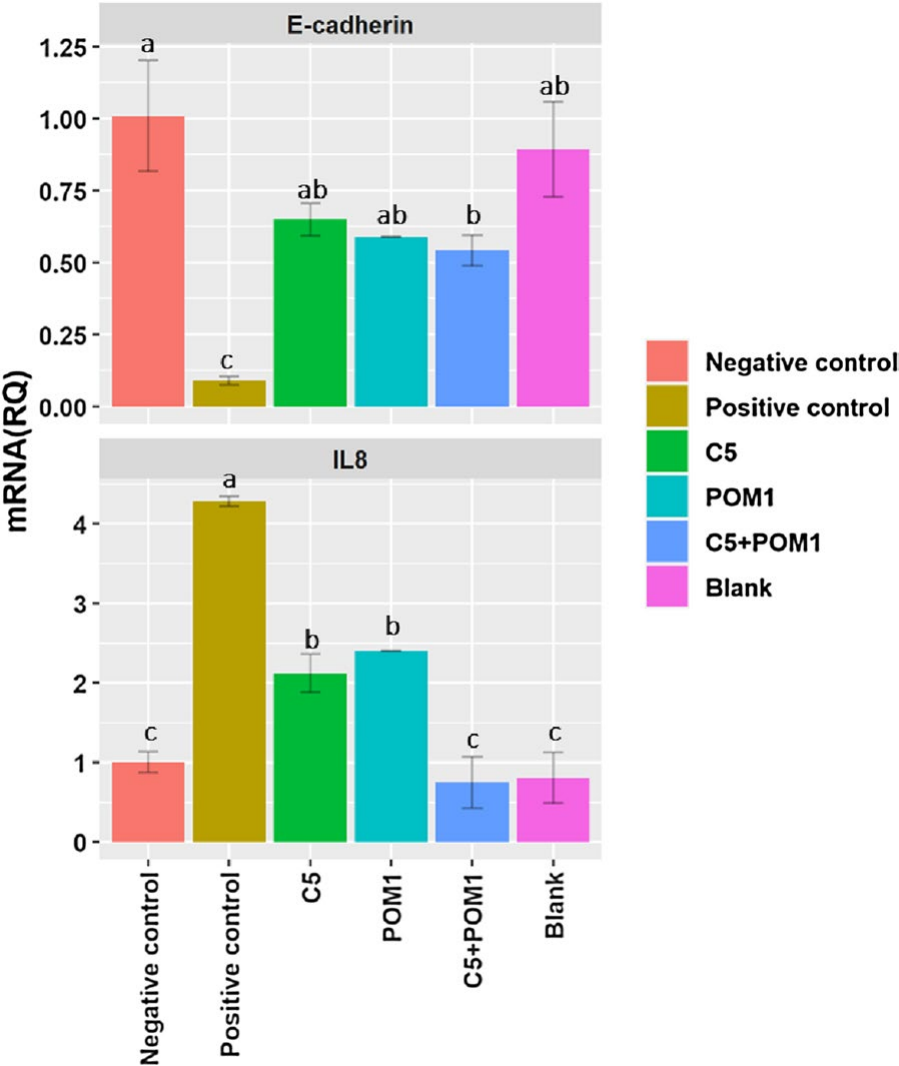
Expression of *E-cadherin* and *Interleukin 8* (mRNA Relative Quantification) in the Human Vaginal Epithelium. Expression rates were calculated as the relative quantification (RQ) data. The data represent mean ± standard deviation. [**a**–**c** Means within the same treatment with different letters are significantly different (P < 0.05)]

HVEs showed a significant decrease (P < 0.05) in interleukin 8 (IL-8) expression, compared to the positive control when HVE models were treated with gels containing the single lactobacilli strains. In addition, a synergistic effect was found when HVEs were treated with the gel containing both lactobacilli strains, also showing a significant difference from the control. A positive effect of the treatment with the gels containing the lactobacilli was visible also from the analysis of the *E-cadherin* expression. The *E-cadherin* expression, which sharply decreased in the HVEs infected with *C. albicans* UNIBZ54, increased when the epithelium model was treated with a gel containing lactobacilli, both in single and in combination (P < 0.05). The results of the gene expression analyses of HVE infected with *C. albicans* UNIBZ54 and treated with a gel containing selected lactobacilli were shown in Fig. 4. The negative control represents the gene expression in the untreated epithelium model, and it is standardized as equal to 1. Under-expression or over-expression of the genes are represented by lower or higher values, respectively. The blank sample is the epithelium model treated with blank gel, the positive control is the epithelium infected with *C. albicans*, while the last three are epithelium models infected with the pathogen and treated with the gel containing the respective strains (*L. plantarum* C5 or *L. plantarum* POM1), or the combination of the two (C5 + POM1).

## Discussion

A healthy human vaginal microbiome is dominated by homofermentative *Lactobacillus* spp., which reflects vaginal community states [23, 24]. The choice of optimum probiotic strains for therapeutic use is crucial since their use can lessen the possibility of the rapid growth of non-native lactobacilli in the urogenital tract in the vaginal environment [25]. The lactobacilli group is the major source of probiotic strains because of their professed benefits on human health [26], and because many of them have been granted the status of qualified presumption of safety (QPS) by the European food safety authority (EFSA) [27]. The vaginal epithelial barrier is shielded from pathogen colonization and invasion by lactobacilli, which produce antibacterial chemicals including lactic acid and hydrogen peroxide, stick to vaginal epithelial cells to form a protective film, and block pathogens’ adhesion [25]. In this study, we screened 95 nomadic lactobacilli isolated from different sources using a multistep approach, for desirable metabolic traits to maintain and restore vaginal health. *L. plantarum* POM1 isolated from tomatoes was the highest producer of lactic acid and d-lactate. *Lact. paracasei* spp. produced significantly lower amounts of lactic acid. This might be because *Lact. paracasei* spp. only synthesizes L-lactic acid isomer since it lacks the gene coding for d-lactate dehydrogenase [28]. The d-lactate production within the vaginal microbiome suppresses the vaginal extracellular matrix metalloproteinase inducer, adding another level of protection against upper genital tract infections [29, 30]. Analogously, most of the native vaginal lactobacilli (*Lb. gasseri, Lb. crispatus,* and *Lb. jensenii*) produce both isomers of lactic acid. Therefore, we favored total and D-lactic acid producers in our scoring procedure. The highest production of H_2_O_2_, a key factor in maintaining the balance of a healthy vaginal environment, was observed for *Lact. paracasei* spp*.*, all isolated from dairy, fruits, and vegetables. Auto-aggregation is suggested to be necessary for the adhesion of probiotic microorganisms to the intestinal epithelium. Both auto-aggregation and hydrophobicity characteristics can be considered a pre-test for selecting probiotic strains which potentially adhere to epithelial cells [31]. *L. plantarum* D9.46 showed the highest auto-aggregation capacity, while *L. plantarum* DM was the only strain showing none. No common characteristics among the strains were observed for both auto-aggregation and hydrophobicity. *S. agalactiae* (GBS) causes an important life-threatening infection in infants, which is transmitted during pregnancy [7, 32]. On the other hand, *C. albicans* is an opportunistic pathogen, which can overgrow in the vaginal ecosystem causing VVC [33]. Given the importance and prevalence of these vaginal pathogens, we assessed the capacity of nomadic lactobacilli CFSs to inhibit them. To exclude pH-dependent inhibition we also included neutralized CFSs. The pathogens' growth inhibition differed substantially across the lactobacilli and pathogens examined [34]. Our findings suggest that the studied *Lactobacillus* strains showed inter-strain differences in anti-*Candida* activity. *Lactobacillus* strains that produce the highest quantities of lactic acid have the strongest antagonistic effect on GBS [35]. These findings imply that *S. agalactiae* is sensitive to organic acids and that individual lactobacilli's capacity to acidify is critical for anti-GBS action. In fact, different pH conditions (original acid or neutral pH) affected the antimicrobial activity against *S. agalactiae*, regardless of the antimicrobial compounds produced (e.g., lactic acid). Microecological preparations, when administered orally or vaginally, have been shown in numerous clinical studies to significantly lower incidence and recurrence rates, prolong the time between recurrences, increase recovery rates, relieve symptoms, and enhance the vaginal microecological patterns of BV and VVC [25]. More in general, some lactobacilli adhere to the vaginal epithelial cells, while others can prevent uropathogens’ attachment to these cells and suppress their growth. Indeed, certain *Lactobacillus* strains can restrict the growth of vaginal pathogens and excrete chemicals that prevent them from multiplying, which are two crucial phases in the pathogenesis of urinary infections [34]. The biofilm formation by lactobacilli is responsible for their stable maintenance of a stable ecosystem, as it grants long-term permanence to the host’s vaginal mucosa [36]. The accessory genome of *Lb. crispatus* contains genomic islands that encode enzymes involved in EPS biosynthesis [37]. In fact, this species is associated with the healthiest vaginal community state [23]. Hence, we determined the  biofilms’ relative composition of protein, eDNA, and total saccharides components. As a result, all the eight previously selected strains were able to form biofilms with distinct macrostructure and composition, which highly diverged between *L. plantarum* strains and *Lact. paracasei* 41j. The main component of ECM is EPS, for all the eight strains, with significant differences (P < 0.05) observed among the strains. We further selected *L. plantarum* POM1 and C5 due to their differentiation from other *L. plantarum* strains (Fig. 1E), and also due to their high biomass production and quantity of EPS (Table 3), respectively, for further investigation. Topical gel formulations containing the two selected strains, in single and in combination, were formulated as a prototype method to be applied to the vaginal surface, using the HVE in vitro model previously infected with *C. albicans* UNIBZ54. *C. albicans* endocytosis has been linked to the proteolytic breakdown of E-cadherin which is the major protein in vaginal epithelial cell junctions [38]. Compared to negative control, infection with *C. albicans* UNIBZ54 decreased the HVEs cell viability up to 25%, while significant (P < 0.05) higher metabolic activity was found when treating HVEs with the topical gel. HVE treated with the gel showed an improved cell viability compared to the untreated one, due to an antagonistic effect of lactobacilli against *C. albicans* UNIBZ54, as already demonstrated by Allonsius et al. [39]. The positive role of the two selected strains was confirmed by the expression analysis of genes encoding for anti-inflammatory cytokines in HVEs. Common vaginal pathogens, including *C. albicans* have been previously associated with elevated expression of IL-8, a proinflammatory chemokine capable of attracting neutrophils to sites of infection [40, 41]. IL-8 gene was overexpressed in cells infected with *C. albicans* UNIBZ54, while the cells treated with the gels containing lactobacilli showed a statistically significant decrease in IL-8 gene expression. A synergistic effect was observed when the cells were treated with the gel containing the combination of the two *L. plantarum* strains. On the other hand, the treatment with the gels containing the two *L. plantarum* strains, both in single and in combination, prevented the reduction of *E-cadherin* gene expression normally caused by the pathogen.

## Conclusions

To summarize, we highlighted the potential of nomadic lactobacilli isolated from different foods, animal, and human sources as suggested probiotic strains for the vaginal environment, thanks to their functional properties and their inhibitory activity against vaginal pathogens (*C. albicans* UNIBZ54 and *S. agalactiae* 88II3). This investigation was carried out to find the isolates with most of the qualities required for *Lactobacillus* to operate as biotherapeutic microorganisms. By using a cumulative scoring-based approach, the pool of potential probiotic candidates was narrowed down. In the end, the ability to form biofilms led to the selection of two *L. plantarum* strains. We provided in vitro evidence on the use of a topic gel containing the two selected potential probiotic strains that can reduce the inflammation caused by *C. albicans* UNIBZ54 infection in the HVE model, either alone or in combination, and thus appear to be promising probiotic strains for vaginal health. Nonetheless, comprehensive clinical investigations will be required to confirm their actual therapeutic advantages.

## Methods

### Bacterial cultures and growth conditions

For this study, a total of 95 lactobacilli were investigated; 48 strains were isolated from dairy products, 31 from fruits and vegetables, 15 from sourdough, and 1 from other sources (Table 5). These strains, together with the pathogenic strain *S. agalactiae* 88II3 and *C. albicans* UNIBZ54, belong to the Micro4Food collection from the University of Bolzano-Bozen. All the cultures were maintained as frozen stocks at − 20 °C in their specific broth medium with 20% glycerol for subsequent analysis. Before their use, the lactobacilli were propagated twice in MRS broth (Sigma Aldrich) at 37 °C (body temperature) for 24 h. *C. albicans* UNIBZ54 was refreshed in Sabouraud broth (Scharlau, Spain) with 5% Tween®80 (Sigma Aldrich) and incubated overnight at 37 °C. *S. agalactiae* 88II3 was refreshed in Brain–Heart Infusion (BHI) broth and incubated at 37 °C until it reached the stationary phase (ca. 24 h).

**Table 5:** Lactobacilli strains (n = 95) used in this study

Number of strains	Species	Source and sub-source of isolation
50	*Lacticaseibacillus paracasei*	
	31a, 25h, 100i, 104g, 22e, 25e, 25g, 28g, 41j, 45j, 50a, 52i, 76d, 83e, 84f, 93j, 99a, AAI9, AII8, BBII10, bI5, dII1, eI3, GII3, HHI10, iiII4, iiII9, JJI8, kI12, LII1, MMII7, wI10, WWI9, zzI10, zzI3, zzI4, zzI6	Dairy (Milk)
	AFI10, AFI7, AFII5, ALII8	Fruits and Vegetables (Apple-by-products)
	S4d8	Fruits and Vegetables (Sauerkraut)
	F1, F10, F12, F13, F2, F23, F25, F5	Dairy (Cheese)
3	*Lactiplantibacillus pentosus*	
	D2.15, E1.4, E3.10	Sourdough
40	*Lactiplantibacillus plantarum*	
	P1, CB5	Dairy (Cheese)
	11j	Dairy (Milk)
	C5	Fruits and Vegetables (Carrot)
	CIL6	Fruits and Vegetables (Cherry)
	Fin6, Fin10	Fruits and Vegetables (Fennel)
	IT1, IT5	Fruits and Vegetables (Grape)
	K1, K13, K2, K9, KI-5	Fruits and Vegetables (Kiwi)
	OE1	Fruits and Vegetables (Olives)
	P3	Fruits and Vegetables (Papaya)
	1LS16	Fruits and Vegetables (Pineapple)
	PR14, PR3, PR6	Fruits and Vegetables (Prune)
	S6w5, AFI5	Fruits and Vegetables (Sauerkraut)
	POM1, POM20, POM27, POM35, POM42, POM43, POM40	Fruits and Vegetables (Tomato)
	DM	Other
	E3.13, E3.19, D9.30, D9.40, D9.46, D3.15, C5.10, D9.18, E3.8, D2.6	Sourdough
2	*Lacticaseibacillus rhamnosus*	
	B6.19, B4.2	Sourdough

### Cell-free supernatants collection

*Lactobacillus* strains were grown in MRS broth for 24 h at 37 °C, and then the CFSs were recovered by centrifugation (7500 rpm, 10 min). Further, CFSs were fractionated in two aliquots. One aliquot was neutralized at pH 7.0 (n-CFSs) with NaOH and sterilized by using a 0.22 μm filter. The other aliquot was preserved at the original acidic pH of ca. 4.0 (o-CFSs) and was further divided into two aliquots: one was sterilized by using a 0.22 μm filter, and one was kept not sterile. All the aliquots of CFSs were stored at − 20 °C until needed. Sterile CFSs were used for pathogen inhibition screening, while non-sterile CFSs were used for functional assays (H_2_O_2_ and lactic acid quantification).

### Lactic acid isomers and hydrogen peroxide quantification

The quantification of lactic acid and H_2_O_2_ in the CFSs was done using two commercial kits, respectively the Megazyme d-Lactate and l-Lactate Assay Kit (Megazyme International Ireland Ltd., Wicklow) and the Peroxide assay kit (Sigma Aldrich), following the instructions provided by the manufacturers.

### Auto-aggregation capacity and hydrophobicity characterization

The candidate strains were grown in MRS at 37 °C for 18–22 h. The pellets were harvested by centrifugation (7500 rpm, 10 min, 5 °C), washed with saline solution (0.9% NaCl), and re-suspended in the same buffer. Cell suspensions were adjusted to an optical density (OD) of 620 nm of ca. 0.25 and used for both analyses (*A*_*0*_ and *H*_*0*_). Cell auto-aggregation was performed according to Gil-Rodríguez, Carrascosa, and Requena [42] as modified by Di Cagno et al. [43]. Each cell suspension was left to settle at room temperature, then the OD_620_ was measured after 2, 4, and 24 h (A_t_). The percentage of auto-aggregation (*A*) was calculated according to the following formulae:

$$A=\left(1-\frac{At}{A0}\right)\times 100$$

Estimation of hydrophobicity was performed according to Burns et al. [44] as modified by Di Cagno et al. [43]. Xylene was used to determine the hydrophobicity of the cell surface. A total volume of 0.4 mL of xylene (Sigma Aldrich) was added to the cell suspension (2 mL), and vortexed for 120 s. After phase stabilization and separation (1 h, 37 °C), the OD of the aqueous phase was measured at 620 nm (Ht). The percentage of hydrophobicity (*H*) was calculated according to the following formulae:

$$H=\frac{\left(H0-Ht\right)}{H0}\times 100$$

### High-throughput pathogen inhibition screening

Growth inhibitions of *C. albicans* UNIBZ54 and *S. agalactiae* 88II3 by CFSs, previously collected and stored, were screened. Briefly, overnight cultures of the pathogens were washed twice with saline solution and resuspended in saline solution to a final OD_600_ of 0.1 for *C. albicans* UNIBZ54, or OD_620_ of 0.25 for *S. agalactiae* 88II3. The 96-well plates were set using one volume of CFSs and three volumes of each pathogen. The analysis was done in triplicate using CFSs previously neutralized (pH 7.0) and the acidic CFSs (pH ca. 4.0). Control wells were prepared using MRS medium at pH 4.0, and MRS at pH 7.0, and run in triplicates. Control MRS was acidified using a racemic solution of lactic acid to pH 4.0 and adjusted back to pH 7.0 using NaOH 1 M to exclude the osmolyte effect. Growth kinetics of the pathogens were recorded for 46 h at 37 °C measuring the absorbance (at a wavelength of 600 nm for *C. albicans* UNIBZ54*,* and 620 nm for *S. agalactiae* 88II3) every 15 min with the Infinite® M Nano + Spectrophotometer (TECAN, Austria).

### Candidate selection approach and scoring procedure

The best-performing lactobacilli were selected through a scoring procedure based on the results of the previously mentioned tests and considering the measure of central tendency along with quartiles. Strains were considered “positive” if the value of each strain was higher than the third quartile (e.g., a value ≥ 25% of the highest values in the dataset for one assay).

$$Score= \frac{\sum\;positive}{total\;number \;of\;essays} \times 100$$

Strains were given a score according to their relative performance on each functional assay: score 1 if Q2 < X > Q3 and score 2 if X > Q3.

Double hits (DH = 1 point) of inhibition by the n-CFSs against *S. agalactiae* 88II3 (SA) or *C. albicans* UNIBZ54 (CA) corresponded to a significant effect (P < 0.05) towards a given pathogen detectable for two or more growth parameters. Strains whose n-CFSs at pH 7.0 showed a double hit (DH) inhibition and o-CFSs that showed a single inhibition effect (H) on *A* at pH 4.0 were scored according to the following calculation:

$$Inhibition\; Score =\left(4\times {DH}_{CA}+ {DH}_{SA}\right)+({4 \times H}_{CA}+ {H}_{SA})$$

Double hit (DH) or hit (H) on *A* for *C. albicans* UNIBZ54 were multiplied by 4 since the frequency of a double hit of inhibition on *C. albicans* UNIBZ54 was ca. 4 times lower than the one on *S. agalactiae* 88II3*.*

### Biofilm preparation

Overnight planktonic cultures of the lactobacilli were centrifuged (7500 rpm, 10 min, 4 °C) and the pellets from each strain were collected and washed twice in saline solution. The suspensions were diluted to an OD_620_ of ca. 0.25 with saline solution. One 5-μL drop of diluted culture was used to inoculate individual sterile membrane filters (pore size, 0.22 μm, Whatman) resting on MRS agar Petri dishes. The membranes were sterilized by UV exposure (15 min per side) before inoculation. The plates were inverted after the inoculum and incubated at 37 °C, with the membrane-supported biofilms transferred to a fresh MRS agar Petri dish every 8 to 10 h [45]. Five membranes were prepared for each strain.

### Biofilm imaging by confocal laser scanning microscopy

One membrane-supported biofilm for each strain culture was visually inspected and photographed by CLSM (Leica SP8LIA, Leica Microsystems). The membrane-supported biofilms were carefully mounted on glass slides. Bacterial cells and the polysaccharide fraction of ECM were stained with 15 μM SYTO® 9 (Invitrogen) and 200 μg mL^−1^ Texas Red®-labeled Concanavalin A (ConA, Invitrogen, stock solution, 5 mg mL^−1^ in 0.1 M sodium bicarbonate) solution in PBS (pH 7.5). Samples were incubated under dark conditions for 1 h at room temperature. Then, biofilm images were collected with a CLSM with excitation at 488 nm and emission > 552 nm lasers. Fluorescence emission was observed between 500–565 nm (for SYTO® 9) and 565–645 nm (for ConA). Images were captured with a 40 × lens using immersion oil and analyzed with the software LAS X (Leica).

### Biofilm growth and ECM characterization

At the end of the incubation, two membrane-supported biofilms for each strain were inserted in separate falcon tubes containing saline solution (9 mL) to quantify the biomass (mg). Cells were then detached and suspended through the vortex (1 min at maximum speed) and serially diluted. Dilutions were plated on MRS agar Petri dishes and the cell density (Log colony forming unit [CFU] mL^−1^) was enumerated after 48 h of incubation at 37 °C. The ECM components of the biofilm colonies were collected and characterized following the method described by Chiba et al. [46]. Briefly, the biofilm colonies were scraped from the membrane filters and suspended in NaCl solution (1.5 M). The suspensions were centrifuged (5,000 g, 10 min, 25 °C) and the supernatants were collected as ECM fractions for the quantification of proteins, total saccharides, and eDNA. The concentration of protein was measured following the Bradford assay [47], using bovine serum albumin (BSA) as a standard. The protein concentration was measured at 590 nm with the UV-1800 Spectrophotometer (SHIMADZU). The total saccharide concentration in the ECMs was measured by the phenol sulfuric acid method [46], using glucose as a standard. Briefly, the isolated ECM fractions (20 μL) were mixed with 5% phenol (20 μL) in a 96-well plate, before adding sulfuric acid (100 μL). The plate was then incubated for 10 min at room temperature, and then the absorbance was read at 492 nm with an Infinite® M Nano + Spectrophotometer (TECAN, Austria). The concentration of the eDNA in the ECM fractions was measured with NanoDrop 2000 (Thermo Fisher Scientific, Waltham, MA, USA).

### Gel formulation

From the results of the second screening, *L. plantarum* C5 and POM1 were selected to be inserted in gel formulations that were developed in collaboration with the R&D Innovation center of Giuliani S.p.A. (Milan, Italy). The cell densities of overnight cultures of the selected lactobacilli were calculated by measuring the OD_620_ using the 6715 UV/VIS Spectrophotometer (Jenway, UK). A final pellet of ca. 11 Log CFU mL^−1^ was collected, then resuspended in 10 mL of vegetal glycerol (Acef s.p.a., Italy), to a final cell density of ca. 10 Log CFU mL^−1^. The glycerol suspension containing lactobacilli was then inserted in an aqueous-based gel formulation. The final formulation contained deionized water (77%), vegetal glycerol suspension (20%) (Solagum tara, Seppic, France), containing lactobacilli (2%), and EUXYL K712 (1%), a liquid cosmetic preservative, containing sodium benzoate and potassium sorbate. The final cell density of the lactobacilli in the probiotic gel was about 9 Log CFU mL^−1^. The viability of the lactobacilli in the gel was assessed by preparing serial dilutions and by plating these on MRS agar medium before any treatment. A total of four gels were prepared, one for each strain previously selected, *L. plantarum* C5 and POM1, one with the combination of the two strains, and one with no cell suspension.

### Human Vaginal Epithelium infection and treatment

The SkinEthic™ HVE model was obtained from EpiSkin (Lyon, France). The HVE model is based on the vulvar epidermoid carcinoma cell line A431 cells which form a 3-D tissue like the human in vivo vaginal mucosa when cultivated in vitro on a polycarbonate filter in a chemically defined medium [48]. After the arrival of the HVEs, the inserts were placed in a 24-well plate, containing maintenance medium (SkinEthic, Episkin) (1 mL), and re-equilibrated for 24 h at 37 °C, in a humidified, 5% carbon dioxide (CO_2_) atmosphere. The HVEs were inoculated with *C. albicans* UNIBZ54 previously adjusted to an OD_600_ ca. 6 Log CFU mL^−1^ using a maintenance medium (SkinEthic, Episkin). The HVEs were previously incubated with *C. albicans* UNIBZ54 (30 µL) in a humidified, 5% CO_2_ atmosphere, at 37 °C for 24 h, to allow the pathogen adhesion and infection. At the end of the incubation period, the HVEs were treated with the gel formula previously prepared (within 24 h from the moment of preparation): two gel formulations containing the single strains of *L. plantarum* C5 and POM1, one gel containing the combination of these two strains, and one gel not containing any strain. Also, some HVEs were left untreated, while HVEs infected with *C. albicans* UNIBZ54 were used as a positive control. After the analysis, the inserts were all incubated for the same time and in the same conditions previously described.

### The MTT assay for cell viability determination

The cell viability of HVEs after infection with *C. albicans* UNIBZ54 and treatment with the gels were determined by the MTT assay [49]. Briefly, the yellow water-soluble salt is reduced by mitochondrial dehydrogenases to purple water-insoluble formazan, according to the viability of cells. A final formazan extraction step is required, using an organic solvent (e.g., isopropanol). A stock solution of MTT (5 mg mL^−1^ in PBS) was diluted (1:10) in the cell culture medium to prepare the MTT solution. The MTT assay was assessed using untreated HVEs (negative control), HVEs treated with blank gel (blank), HVEs infected with *C. albicans* UNIBZ54 (positive control), and HVEs infected with *C. albicans* UNIBZ54 and treated separately with *L. plantarum* C5 and POM1, and with the gel containing a combination of these two strains. After 24 h of incubation, the inserts were rinsed with PBS and placed in a new 12-well plate containing MTT solution (300 μL). After 2 h of incubation (humidified, 5% CO_2_ atmosphere, 37 °C), isopropanol (800 μL) was added to each well. The plate was incubated again for 1.5 h, in the same conditions above mentioned. Two aliquots (200 μL) were then taken from each well and placed in 96-well plates. The OD_570_ was measured with a BioTek Micro-volume Plate Reader (BioTek Instruments Inc., Bad Friedrichshall, Germany) and elaborated with the ELX808 software (BioTek Instruments Inc., Bad Friedrichshall, Germany) (reference filter: 630 nm). Results were expressed as a percentage of viability compared to the negative control (mean ± standard error of triplicate cultures), using the following formula:

$$\mathrm{Viability }(\mathrm{\%}) = [{\mathrm{OD}}_{(570\mathrm{ nm}-630\mathrm{ nm})}\mathrm{ test product }/ {\mathrm{OD}}_{(570\mathrm{ nm}-630\mathrm{ nm})}\mathrm{ negative control}] \times 100$$

### Gene expression profiling

The total RNA was extracted from the HVEs, previously infected with *C. albicans* UNIBZ54 and treated with the gels, using the RNeasy mini kit (Qiagen, Valencia, CA, USA), according to De Vuyst [50]. Briefly, the circumference of the polycarbonate filter was dissected from the bottom of the insert using a sharp surgical blade and then transferred into a 12-well culture plate containing RLT buffer (600 μL). After 1 or 2 min, the stratum corneum, detached from the epidermis, was removed using a pair of tweezers and discarded. For disrupting keratinocytes, the epidermis was gently scratched with a micropipette tip in the lysis buffer, provided by the kit. The lysate was homogenized by pipetting and then transferred into a spin column placed into a 2.0 mL collection tube. This procedure allows the recovery of enough RNA from the HVEs for real-time (RT) polymerase chain reaction (PCR) analysis of gene expression. Following the instructions provided by the manufacturer, 2 μg of RNA templates were used to synthesize complementary DNA (cDNA) in a 20 μL reaction volume, using the *PrimeScriptTM RT Reagent Kit* (TakaraBioInc., Japan). The cDNA was amplified and detected by the Stratagene Mx3000P RT-PCR System (Agilent Technologies Italia S.p.A., Milan, Italy). PCR conditions were the following: 37 °C for 15 min, 85 °C for 5 s, and 25 °C for 2 min. Afterward, the TaqMan® Gene Expression Assays were carried out for RT-PCR using the following genes: the glyceraldehyde-3-phosphate dehydrogenase (GAPDH) Hs99999905_m1, IL-8 Hs00174103_m1, the cadherin 1 (CDH1) Hs01023894_m1. The GAPDH was used as a housekeeping gene. PCR amplifications were carried out in 20 µL of total volume. The mixture of reaction contained 10 µL of 2× Premix Ex Taq (Takara, Japan), 1 µL of 20× TaqMan Gene Expression assay, 0.4 µL of RoX Reference Dye II (Takara, Japan), 4.6 µL of water, and 4 µL of cDNA.

### Data and statistical analysis

All the analyses were performed considering three biological replicates analyzed in triplicate. The growth parameters of each pathogen were determined using the grofit R package [51] for each replicate of CFSs and controls. Growth parameters were determined using the free splines approach and bootstrapping with 100 resamplings. The inhibition effect was assessed through a non-parametric one-way Kruskal–Wallis followed by the Dunn Control post hoc test provided by the PMCMR R package [52]. The resulting p-values were adjusted for multiple hypothesis testing using Benjamini and Hochberg false discovery rate correction (FDR). Data from the determination of biofilm formation were submitted for analysis of variance by the General Linear Model (GLM) of R statistical package (R, version 1.6.2 rcompanion.org/handbook/). Multi-comparison of treatment means was achieved by a Tukey-adjusted comparison procedure with a p-value < 0.05 [53]. For the gene expression profiling, the average value of the target gene was normalized using the GAPDH gene, and the relative quantification of the levels of gene expression was determined by comparing the Δ cycle threshold (ΔC_t_) value [54]. The statistical analysis was performed using GraphPad Prism 6 (GraphPad Software Inc). Data are expressed as the mean, mean ± standard error of the mean (SEM), or mean fold change ± SEM. p-values < 0.05 were considered as statistically significant.

## Data Availability

The datasets used and/or analyzed during the current study are available from the corresponding author upon reasonable request.

## Abbreviations

BHI: Brain Heart Infusion
BSA: Bovine serum albumin
BV: Bacterial vaginosis
CDH1: Cadherin 1
cDNA: Complementary DNA
CFS: Cell-free supernatant
CFU: Colony Forming Unit
CLSM: Confocal laser scanning microscopy
CO_2_: Carbon dioxide
ConA: Concanavalin A
CST: Community state type
ECM: Extracellular matrix
eDNA: Extracellular DNA
EFSA: European Food Safety Authority
EPS: Exopolysaccharides
FDA: Food and Drug Administration
FDR: False discovery rate
GBS: Group B *Streptococcus*
GAPDH: Glyceraldehyde-3-phosphate dehydrogenase
GRAS: Generally recognized as safe
H_2_O_2_: Hydrogen peroxide
HVE: Human vaginal epithelium
IL-8: Interleukin 8
MRS: De-Man-Rogosa-Sharpe
MTT: 3-(4,5-Dimethylthiazole-2-yl)-2, 5-diphenyl tetrazolium bromide
OD: Optical density
PCR: Polymerase chain reaction
PBS: Phosphate buffer solution
QPS: Qualified Presumption of Safety
RT: Real-Time
SEM: Standard error of mean
VVC: Vulvovaginal candidiasis
